# Genomic analysis of high copy-number sequences for the targeted detection of *Listeria* species using a flow-through surveillance system

**DOI:** 10.1007/s00203-021-02388-2

**Published:** 2021-06-02

**Authors:** Beatriz Quiñones, Jaszemyn C. Yambao, Veronica S. De Guzman, Bertram G. Lee, David L. Medin

**Affiliations:** 1 U.S. Department of Agriculture, Agricultural Research Service, Western Regional Research Center, Produce Safety and Microbiology Research Unit, Albany, CA 94710 USA; 2 SnapDNA, Inc, Mountain View, CA 94043 USA

**Keywords:** *Listeria*, Food safety, Fresh produce, RNA, Foodborne pathogen, Genomes

## Abstract

The bacterial foodborne pathogen *Listeria monocytogenes* has been implicated in fresh produce outbreaks with a significant economic impact. Given that *L. monocytogenes* is widespread in the environment, food production facilities constantly monitor for the presence of *Listeria* species. To develop a surveillance platform for food processing facilities, this study conducted a comparative genomic analysis for the identification of conserved high copy sequences in the ribosomal RNA of *Listeria* species. Simulated folding was performed to assess RNA accessibility in the identified genomic regions targeted for detection, and the developed singleplex assay accurately detected cell amounts lower than 5 cells, while no signals were detected for non-targeted bacteria. The singleplex assay was subsequently tested with a flow-through system, consisting of a DNA aptamer-capture step, followed by sample concentration and mechanical lysis for the detection of *Listeria* species. Validation experiments indicated the continuous flow-through system accurately detected *Listeria* species at low cell concentrations.

## Introduction

Foodborne pathogens are responsible for a consistent level of human illness that poses a substantial public health and economic burden, resulting in an economic burden of $15.5 billion annually (Hoffmann et al. 2015). Listeriosis, a serious infection caused by eating food contaminated with the bacterium *Listeria monocytogenes*, has been recognized as an important public health problem in the United States, and the annual economic impact of listeriosis in the United States alone is estimated at over US$2.8 billion. There has been an estimated 1500 listeriosis cases each year, and of those, approximately 300 cases have resulted in death (Hoffmann et al. 2012; Scallan et al. 2011). In particular, *L. monocytogenes* is responsible for systemic listeriosis with an approximate 30% mortality rate in susceptible populations of pregnant women, neonates, elderly, or immunocompromised patients (Buchanan et al. 2017; Hoffmann and Scallan Walter 2019; Scallan et al. 2011).

While *L. monocytogenes* is considered one of the major foodborne pathogens that cause listeriosis in humans and animals (Pesavento et al. 2010), other *Listeria* species, which include *Listeria grayi, Listeria innocua, Listeria ivanovii, Listeria seeligeri, and Listeria welshimeri,* have been occasionally implicated in human clinical case reports, primarily in individuals with suppressed immune functions and/or underlying illnesses (Korsak and Szuplewska 2016). *Listeria* species are widely distributed in various environments, including soil, water, vegetation, animal feed, farm environments, food processing environments, urban and suburban environments (Korsak and Szuplewska 2016). *L. monocytogenes* and *L. innocua* have been the most prevalent species of *Listeria* found in urban environments, produce production, preharvest environments, retail environments, and processing environments (Estrada et al. 2020). Recently, high profile outbreaks of *L. monocytogenes* have been associated with deli meats, fresh produce, and ready-to-eat foods (Churchill et al. 2019). Other *Listeria* species have also been found in ready-to-eat, raw or unprocessed foods (Arslan and Özdemir 2020; Guerra et al. 2001; Pesavento et al. 2010; Soriano et al. 2001; Zeinali et al. 2017), as well as in food processors (Huang et al. 2007; Korsak and Szuplewska 2016) and other food products (Chen et al. 2009; Pesavento et al. 2010). Given that *L. monocytogenes* is widespread in the environment, food production facilities constantly monitor and control for the presence of *Listeria* species on surfaces. *Listeria* species are considered a broad indicator of the conditions potentially favorable for *L. monocytogenes* growth and survival in the environment (Brouillette et al. 2014; The United Fresh Food Safety & Technology Council 2018; Zoellner et al. 2018). Using a broad indicator group, such as screening for all *Listeria* species (genus level), increases the chances of finding *L. monocytogenes* niches and reacting in an effective manner to mitigate the prevalence of this pathogen in a food production facility (Brouillette et al. 2014; The United Fresh Food Safety & Technology Council 2018; Zoellner et al. 2018).

Given that conventional culture-based assays are labor intensive and time consuming, several methodologies have been developed for the identification of foodborne pathogens, which can be further classified into nucleic acid-based, biosensor-based and immunological-based methods (Chen et al. 2017; Law et al. 2015). In particular, nucleic acid-based methods, employing real-time quantitative PCR (qPCR), have become preferred methods for the detection and quantification of *Listeria* species due to their simplicity, high sensitivity and specificity, and low risk of contamination due to the lack of post-processing steps for obtaining the detectable signal when compared to conventional PCR (Chen et al. 2017; Gasanov et al. 2005). Recently, several multiplex qPCR assays have become available for the identification of multiple *Listeria* species by targeting genes required for virulence and regulatory functions including those coding for bacteriolytic properties (*iap*), phosphatidylinositol phospholipase C (*plcB*), sucrose-specific enzyme (*scrA*) as well as putative internalin and oxidoreductase, and N-acetylmuramidase proteins (Chen et al. 2017; Hage et al. 2014; Hein et al. 2001; Hitchins et al. 2017). Other qPCR assays have been developed by targeting the 23S ribosomal DNA or non-coding RNA specific to the *Listeria* genus; however, these qPCR assays require multiple sets of primers and probes to be multiplexed to enable the detection of various *Listeria* species (Chen et al. 2017; Petrauskene et al. 2017; Rodríguez-Lázaro et al. 2004).

In the past years, there has been a significant increase in the availability of bacterial genome sequences in public databases (Land et al. 2015; Sayers et al. 2020). These recent developments would consequently enable the improvement of the new design of singleplex qPCR assays for detecting multiple strains of *Listeria* species by allowing better identification of specific and accessible genomic regions to be inclusive in detecting the targeted species while still being exclusive in discriminating against non-*Listeria* strains. Given that ribosomal RNA (rRNA) is present in large quantities and multiple copies in bacteria, in particular in the *Listeria* genome (Glaser et al. 2001; Milner et al. 2001), new approaches on the development of molecular assays for detecting foodborne pathogens have chosen RNA as the targeted analyte (Livezey et al. 2013), which offers a reliable and highly sensitive detection of *Listeria* species. In the present study, a comparative genomic analysis was conducted for evaluating high copy-number sequences to enable the targeted and specific detection of *Listeria* species. By conducting an in silico analysis with a dynamic programming algorithm, simulated folding was performed to assess the accessibility of multicopy targeted regions in the rRNA for optimal detection. This nucleic acid-based assay, a singleplex assay, was further validated by conducting inclusivity tests for *Listeria* species and exclusivity tests for non-targeted environmental bacterial strains, belonging to the *Bacillus*, *Citrobacter*, *Enterobacter*, *Pseudomonas*, *Salmonella* and *Shigella* genera. As a proof-of-concept for the development of an in-process detection system for foodborne pathogens in food processing facilities, this nucleic acid-based assay was tested in conjunction with a flow-through system, consisting of aptamer-capture step, followed by sample concentration and mechanical lysis for the detection of *Listeria* species at low cell concentrations.

## Materials and methods

### Bacterial strains and culture conditions

The *Listeria* strains used in the present study (Table 1) were streaked for isolation on BBL™ Trypticase™ Soy Agar (Becton, Dickinson and Co., Franklin Lakes, NJ) with 0.6% Bacto™ Yeast Extract (Becton, Dickinson and Co.) (Hitchins et al. 2017). *Listeria* species liquid cultures were grown in BD™ Tryptic Soy Broth (Becton, Dickinson and Co., Franklin Lakes, NJ) or Brain Heart Infusion (BHI) Broth (K25; Hardy Diagnostics, Santa Maria, CA) and were further incubated overnight with constant shaking (225 rpm) at 37 °C. Non-targeted bacterial strains (Table 1) were streaked for isolation on Luria–Bertani agar (LB; Becton, Dickinson, and Co.), and liquid cultures were grown in LB broth overnight with constant shaking (225 rpm) incubated at 37 °C for strains belonging to the genera *Citrobacter*, *Escherichia*, *Salmonella*, and *Shigella* and incubated at 28 °C for strains belonging the genera *Bacillus*, *Enterobacter*, and *Pseudomonas*.

**Table 1:** Bacterial reference strains used in this study

Species	Strain	Other designation^a^	Source	Location	Reference or provider^b^
*Bacillus cereus*	RM3190	N/A	Soil	USA	(Friedman et al. 2006)
	RM5141	6A1	Soil	USA	*Bacillus* Genetic Stock Center
	RM5142	6A2	Soil	USA	*Bacillus* Genetic Stock Center
	RM5143	6A3	Soil	USA	*Bacillus* Genetic Stock Center
	RM20582	ATCC 14,579	Soil	USA	(Ivanova et al. 2003)
*Bacillus maroccanus*	RM3191	N/A	Soil	USA	Laboratory Collection
*Bacillus subtilis* subspecies *spizizenii*	RM19627	ATCC 6633	Soil	USA	(Waleh and Ingraham 1976)
*Citrobacter braakii*	RM7359	ATCC 43,162	Human	USA	(Shanks et al. 2012)
*Citrobacter freundii*	RM4680	N/A	Lettuce	USA	Laboratory Collection
*Citrobacter youngae*	RM3642	N/A	Moss	USA	(Bettelheim et al. 1993)
*Escherichia coli*	RM5034	ATCC 29,425	N/A	Basel	(Quiñones et al. 2011)
Enteropathogenic *Escherichia coli* O55:H7	RM13616	99,107 (90)	Human	Brazil	(Kyle et al. 2012)
*Enterobacter asburiae*	RM3638	N/A	Soil	USA	(Cooley et al. 2003)
*Enterobacter cloacae*	RM9194	P-1242	Spinach	USA	(Cooley et al. 2007)
	RM9195	P-1248	Lettuce	USA	(Cooley et al. 2007)
*Listeria grayi*	RM2207	ATCC 19,120	Rodent feces	USA	(Rocourt et al. 1992)
	RM2208	ATCC 25,401	Corn	USA	(Rocourt et al. 1992)
	RM3344	BA54	Ice cream	USA	Laboratory Collection
*Listeria innocua*	RM2209	ATCC 33,090	Cattle	USA	(Rosimin et al. 2016)
	RM2438	ATCC 51,742	Cabbage	USA	(Niemira et al. 2003)
	RM2442	CDHS# 90E01283-L-11	Shellfish	USA	Laboratory Collection
	RM3320	SEA 15b60	Lettuce	USA	Laboratory Collection
*Listeria ivanovii*	RM2206	ATCC 19,119	Sheep	Bulgaria	(Longhi et al. 2014)
	RM3096	ATCC 700,402	N/A	USA	(Charlermroj et al. 2019)
	RM3325	SEA 15a96	Cheese	USA	Laboratory Collection
*Listeria monocytogenes*	RM2199	F2379	Human	USA	(Gorski et al. 2006)
	RM3022	ATCC 19,116	Chicken	England	(Wei et al. 2019)
	RM3106	N/A	Processing plant	USA	(Gorski et al. 2006)
	RM3871	Isolate# 33,123	Floor drain	USA	(Liu et al. 2018)
	RM17178	FN-290–1	Water	USA	(Cooley et al. 2014)
*Listeria seeligeri*	RM2211	ATCC 35,967	Soil	Germany	(Ryu et al. 2013)
	RM3321	SEA 3126	Cheese	USA	Laboratory Collection
	RM3322	SEA 2232	Fish	USA	Laboratory Collection
*Listeria welshimeri*	RM2210	ATCC 35,897	Decaying plant material	USA	(Ryu et al. 2013)
	RM3323	SEA 15b05	Cheese	USA	Laboratory Collection
	RM3324	SEA 3659	Fish roe	USA	Laboratory Collection
*Pseudomonas chlororaphis*	RM2107	FC4	Cilantro	USA	(Brandl and Mandrell 2002)
*Pseudomonas syringae*	RM1952	B728a	Bean	USA	(Feil et al. 2005)
	RM14153	N/A	Lettuce	USA	Laboratory Collection
*Salmonella enterica* serovar Kentucky	RM7890	MH67589	Ground chicken	USA	(Fagerquist and Zaragoza 2018)
Shiga toxin-producing *Escherichia coli* O104:H4	RM14735	11EN0819	Human	Germany	(Cooley et al. 2013)
Shiga toxin-producing *Escherichia coli* O157:H7	RM2084	EDL-933; DEC 4f	Meat	USA	(Quiñones et al. 2012)
*Shigella boydii*	RM7079	6326/9	N/A	Germany	Laboratory Collection
*Shigella flexneri*	RM7078	SEA 13,618	Human	USA	Laboratory Collection
*Shigella sonnei*	RM7077	SEA 136,012	Human	USA	Laboratory Collection

^a^ N/A, Not available

^b^ Laboratory Collection at U.S. Department of Agriculture, Agricultural Research Services, Western Regional Research Center, Produce Safety & Microbiology Unit, 800 Buchanan Street, Albany, CA 94,710 USA. The *Bacillus* Genetic Stock Center, Biological Sciences 556, 484 West 12th Avenue, Columbus, OH 43,210 USA

### Comparative genomics and in silico analysis for oligonucleotide design

To design oligonucleotides targeting the rRNA operon (*rrn* operon), 59 ribosome sequences of 19 *Listeria* species including the common *Listeria* species: *L. innocua*, *L. ivanovii*, *L. grayi*, *L*. *monocytogenes*, and *L. welshimeri. L. monocytogenes* strains with all known serovars were included for the design (Doumith et al. 2004). The ribosome sequence of *L*. *monocytogenes* strain EGD-e (GenBank Accession No. CP023861) was chosen as a model ribosome sequence (Glaser et al. 2001). Non-targeted bacterial sequences were selected by searching the top non-*Listeria* matches with the model ribosome sequence. Additional bacterial strains belonging to a diverse genera such as *Citrobacter*, *Enterobacter*, and *Pseudomonas*, commonly found as resident bacteria in agricultural and food processing environments (Dees et al. 2015; Møretrø and Langsrud 2017; Orsi and Wiedmann 2016; Williams and Marco 2014; Williams et al. 2013), were also added to the non-targeted list of genomes examined. Simulated folding of the model sequence was performed to find regions of high RNA accessibility with unfolding energy < 20 kcal/mol using Visual-OMP™ software package (DNA Software, Inc., Ann Arbor, MI). The target sequences and non-target sequences were aligned using Geneious Software (Biomatters, Ltd., Aukland, NewZealand). Regions of high RNA accessibility in the model ribosome sequence with the most heterogeneity with an average of less than 80% match with the reference sequence were selected as target regions for the primer design using RealTimeDesign™ qPCR Assay Design Software (LGC, Biosearch Technologies, Petaluma, CA). The use of the RealTimeDesign™ software allowed the design with the proprietary BHQplus® probes incorporating a duplex-stabilizing technology (LGC, Biosearch Technologies) to elevate the melting temperature and enhance target specificity (Kutyavin 2008). The default of least restrictive parameters for RealTimeDesign™ software were used in the design (Sowers et al. 2005, 2006); however, for some regions, the default parameters were modified to design oligonucleotides with increased specificity to the model target. In particular, the minimum primer temperature was lowered from 65 °C to 60 °C, and the maximum probe temperature was increased from 72 °C to 77 °C. Additionally, the maximum internal stability was increased to 0.6 from 0.0. In some instances, runs of four guanidine bases were allowed to improve specificity. Three guanidine and/or cytosine bases were allowed at the 3’ end of the oligonucleotide, and the maximum percentage of the guanidine bases in the oligonucleotide probes was increased to 40%.

### Nucleic acid extractions

Purified RNA was extracted from diluted overnight broth cultures using the ClaremontBio RNAexpress with OmniLyse® HL Kit and DNAexpress columns (Claremont BioSolutions, LLC, Upland, CA) or the Qiagen RNeasy® Protect Bacteria Mini Kit (QIAGEN, Inc., Valencia, CA), according to the manufacturer’s guidelines. The quantity of nucleic acid was assessed by fluorometric measurement using the Qubit™ 4 Fluorometer (Invitrogen, Carlsbad, CA), and the quality was evaluated using an Agilent Bioanalyzer 2100 (Agilent Technologies, Santa Clara, CA). Crude lysates and plate enumerations were prepared by aliquoting diluted overnight broth culture at mid-log (OD_600_ absorbance ~ 0.2–0.3) and serially diluting tenfold in nuclease-free water and 1 × phosphate-buffered saline (PBS), respectively. To prepare crude lysates, each serially diluted culture in nuclease-free water was lysed by mechanical disruption using an OmniLyser (Claremont BioSolutions, LLC). For plate enumerations, a 10 µl droplet of each serially diluted culture in 1 × PBS was plated on fresh solid LB agar overnight at 37 °C for *Citrobacter* species, *Escherichia* species, *Listeria* species, *Salmonella* species, and *Shigella* species strains and 28 °C for *Bacillus* species, *Enterobacter* species, and *Pseudomonas* species.

### Nucleic acid amplifications

Using a unidirectional workflow previously employed for experimental procedures with foodborne enteric viruses (Quiñones et al. 2017), the amplification by reverse transcription-quantitative PCR (RT-qPCR) was performed in a 20 µl reaction mixture containing 5 µl of 4 × TaqPath™ 1-Step Multiplex Master Mix (Applied Biosystems, Foster City, CA), 0.5 µM of each forward and reverse BH1 primer, 0.1 µM of BH1 probe (LGC, Biosearch Technologies) and 1 µl purified RNA or 5 µl crude lysate. The reaction mixtures were placed in a QuantStudio™ 5 Real-Time PCR System (Applied Biosystems) with the following parameters: RT step at 54 °C for 15 min, hot start at 95 °C for 2 min followed by 40 cycles each of denaturation at 95 °C for 3 s and annealing and extension at 64 °C for 30 s. Cycle threshold (Ct) values were analyzed using the QuantStudio™ Design & Analysis software (Applied Biosystems). The performance of the BH1 oligonucleotide-based assay was compared with a DNA-based assay, MicroSEQ™ *Listeria monocytogenes* Detection kit (Applied Biosystems) with tenfold serial dilutions of *L. monocytogenes* cell lysate. The estimated cell concentrations in the suspension were determined by optical density and confirmed by viable bacterial colony count on solid media.

### Design of a continuous flow-through detection system

For the design and validation of the continuous flow-through detection system, *L. grayi* strain RM2208 was used as the model organism (Table 1). In detail, samples were blended with capture buffer (0.2 × PBS, 2 mM magnesium chloride, and 0.2% Triton X-100) and filtered into a sample container using a Whirl–Pak® sterile sampling bag with 330 µm filter membrane (B01547; Nasco, Fort Atkinson, WI) to remove particulates. A biocompatible Masterflex™ peristaltic pump (Masterflex™ Model 7550–60; Cole-Parmer Instrument Co., Vernon Hills, IL) introduced the liquid sample into a Tenny Jr. temperature-controlled chamber (Model TLJR; Tenney Environmental, Parsippany, NJ) (Medin et al. 2020). Fluidic valves (Model SMC LVM205R-6B1; SMC Pneumatics, Yorba Linda, CA) moved homogenized sample through a depth filter, consisting of glass beads (100 µm diameter; BioSpec Products, Inc., Bartlesville, OK) having non-fouling surface properties (Medin et al. 2020). The *Listeria* cells were then captured in the column using aptamer-functionalized soda-lime glass beads (100 µm diameter; BioSpec Products, Inc.) with silane surface modification for attachment of the oligonucleotide sequences, serving as a spacer sequence (5′-GTTTTTGTTTTGAAAGTTGTTTTTTTTTT-3′) for aptamer extension and a tether sequence (5′-CAACTTTCAAAACAAAAACTTTTTTTTTT-Amino C6-3′) for aptamer surface attachment (Medin et al. 2020; Quiñones et al. 2020). The release of the sequestered *Listeria* cells from the column using release buffer (1 M NaCl, 100 mM EDTA, 0.1% Tween 20, 0.05% SDS, 30 mM NaHCO3, pH 10.3–10.4) (Medin et al. 2020). After potential inhibitors were removed as waste, the released *Listeria* cells were mechanically lysed using an ultrasonic transducer (Black & Decker, Baltimore, MD) and yttrium-stabilized zirconia beads (100 µm diameter; Pingxiang Chemshun Ceramics Co. Ltd., Pingxiang City, Jiangxi Province, China). An aliquot of the lysate was subjected to further amplification by RT-qPCR by using the BH1 oligonucleotide assay. To determine the capture efficiency of the aptamer-functionalized column, the capture column was conditioned by adding approximately 30 ml of capture buffer through the column at 15 ml/min using a Masterflex™ peristaltic pump (Cole-Parmer Instrument Co.), and the conditioning flow-through was discarded. A 10 ml *Listeria* cell suspension, diluted to a final concentration of approximately 1.6 × 10^5^ CFU/ml, was added to the capture column. The entire flow-through was collected, and the *L. grayi* cell concentration was enumerated. The capture efficiency to the aptamer column was determined as the fraction of *Listeria* cells from the starting cell suspension added to the capture column that were not detected in the flow-through cell count, as previously described (Medin et al. 2020).

### Statistical analysis

Statistical significance in the detected Ct values was determined by performing two-tailed Fisher’s exact test using R statistical software (version 3.0.1; R Foundation for Statistical Computing, Vienna, Austria) (Fisher 1935; Mehta and Patel 1986). Descriptive statistical analyses and one-way analysis of variance (ANOVA) were performed using the Analysis ToolPak in Microsoft® Excel® version 2002 for Office 365 ProPlus (Microsoft Corporation, Redmond, WA). *P*-values of ≤ 0.005 were considered statistically significant.

## Results and discussion

**Design strategy of the oligonucleotide-based assay for detecting** ***Listeria*** **species**

**Fig. 1:**
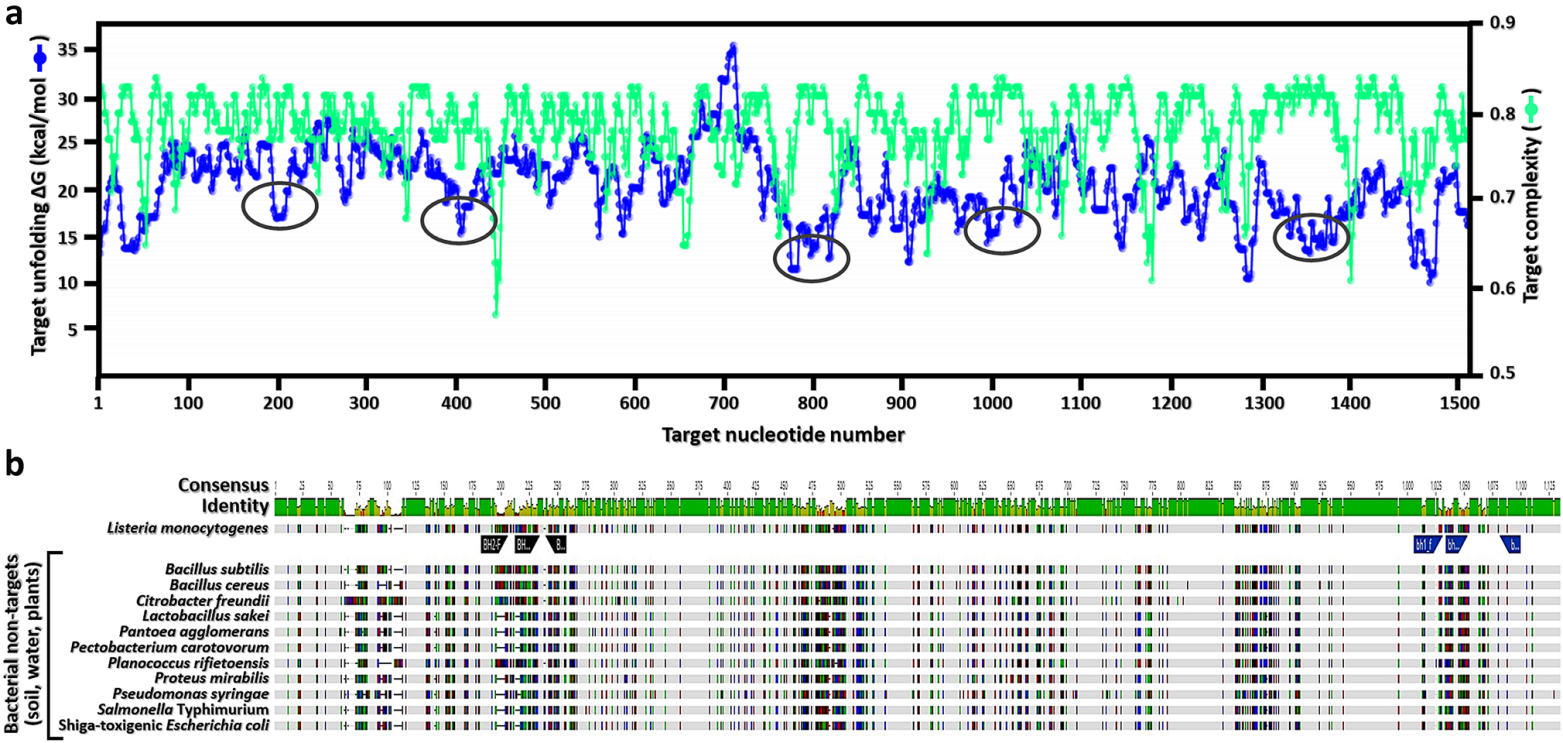
Design strategy and specificity of the oligonucleotide-based assay for detecting *Listeria* species. Ribosomal RNA (rRNA) was chosen as the targeted region to enable a reliable detection of *Listeria* species at low cell concentrations. **a** Simulated folding was performed to assess RNA accessibility in the identified regions (circles) by calculating the equilibrium target unfolding (blue symbols) and the target complexity (green symbols) calculations, as determined using Visual-OMP™ software package (DNA Software, Inc., Ann Arbor, MI). **b** To further assess the oligonucleotide specificity for cross hybridization with non-targeted bacterial species from soil, water, and plant surfaces, the in silico mismatch was examined by comparing *Listeria monocytogenes* sequences with strains belonging to *Bacillus, Citrobacter, Escherichia*, *Lactobacillus*, *Pantoea*, *Pectobacterium, Planococcus, Proteus, Pseudomonas*, and *Salmonella* genera

As a suitable targeted region, rRNA has been considered for the development of detection assays as it is found to be represented in the bacterial genome in multiple copies (Livezey et al. 2013; Milner et al. 2001). In particular, whole-genome analysis revealed that a single *L. monocytogenes* cell contains 6 copies of the rRNA (*rrn*) operon (Glaser et al. 2001), and expression studies estimated that approximately 600–25,000 copies of the ribosomes were detected per cell (Milner et al. 2001). These findings have indicated that targeting rRNA is more than enough for a reliable amplification of nucleic acids to enable detection of the targeted pathogen at low cell concentrations. To initiate the design of oligonucleotides, approximately 200 genomic entries of distinct species of *Listeria* and non-targeted bacterial species were aligned and compared, and regions in the ribosome sequences with the most heterogeneity between the *Listeria* sequences and non-target sequences were selected for the probe design (Fig. 1). The distinct species of *Listeria* for the comparative genomic analysis included *Listeria aquatica, Listeria booriae, Listeria cornellensis, Listeria costaricensis, Listeria goaensis, Listeria fleischmannii, Listeria floridensis, Listeria grandensis, L. grayi, L. innocua, L. ivanovii, Listeria marthii, L. monocytogenes, Listeria newyorkensis, Listeria riparia, Listeria rocourtiae, L. seeligeri, Listeria thailandensis, Listeria weihenstephanensis,* and *L. welshimeri*.

The in silico analyses were performed with algorithms using the nearest-neighbor model coupled with multi-state equilibrium model for conducting simulated folding to assess RNA accessibility in the identified genomic regions targeted for detection (Fig. 1a). The results revealed multiple locations in the genome with an optimal target complexity as well as target unfolding. As shown in Fig. 1a, analysis of the 16S rRNA in *L. monocytogenes* revealed that the targeted region for the oligonucleotide design had an unfolding delta G calculation to be approximately 14 K to 16 K cal/mol when compared to most sites in the examined region with much higher values of 26–28 K cal/mol. To reduce any false positives due to low levels of specificity in the oligonucleotide design, the complexity of the targeted regions was also further examined (Fig. 1a). The analysis of optimal genome locations was further assessed to select the best sites for designing the targeted oligonucleotides (Fig. 1a, circles), and these selected sites for the design of the oligonucleotides were found to have optimal complexity scores between 0.8 and 1, indicating relative accessibility and complexity of regions within the target sequences (Nielsen et al. 2003). Additionally, the in silico mismatch was examined by comparing a collection of various *Listeria* species genome sequences with other bacterial non-targets, commonly present from soil, water, and plant surfaces (Fig. 1b). The sequence alignment to non-targeted bacterial species, included *Bacillus* species*,Escherichia* species, *Pseudomonas* species, and *Citrobacter* species, representing non-targeted bacterial species from agricultural environments (Dees et al. 2015; Orsi and Wiedmann 2016; Williams and Marco 2014; Williams et al. 2013).

To increase the level of desired target specificity at the genus level, the oligonucleotide design targeted the *rrn* operon comprising of the 16S and 23S rRNA gene clusters in the *Listeria* genome, a region commonly used as a microbial molecular marker for phylogenetic classification of bacterial species (Woese 1987). The design employed base analogs to promote nucleic acid duplex stabilizing, enabling an elevated melting temperature of the oligonucleotides (Kutyavin 2008). Additional standard criteria were also employed for developing the nucleic acid amplification assays, which consisted of examining for the G–C content, annealing temperatures, and self-hybridization (Sowers et al. 2005, 2006). The in silico analysis resulted in a total of six oligonucleotide sets with a G–C content ranging from 36.8% to 57.9% and annealing temperatures ranging from 55.4 °C to 76.6 °C (Table 2).

**Table 2:** List of oligonucleotides used in this study

Oligonucleotide name	Sequence (5' → 3')^a^	rRNA target	Amplicon size (bp)	Annealing temperature (C°)^b^	GC%	Ct values^c^	
						10 fg *Listeria*	1 ng *Bacillus*
L1R1-Fwd	GTTACGATTTGTTGCAAGGTTAAGCGGAAA			65.2	40.0%		
L1R1-Rev	ACCTTCATCCTGGACATGGG	23S	126	59.1	55.0%	31.6	32.5
L1R1-Probe	FAM^a^-GCGGAGCCGTAGCGAAAGCG-BHQ1			65.2	40.0%		
BH1-Fwd	CCTTACCAGGTCTTGACATTCTTTG			65.6	44.0%		
BH1-Rev	GAGCTGACGACAACCATGC	16S	93	65.3	57.9%	30.9	No Ct
BH1-Probe	FAM-CACTCTGGAGACAGAGCTTT-BHQ1			68.2	50.0%		
BH2-Fwd	CTAATACCGAATGATAAAGTGTGG			61.1	37.5%		
BH2-Rev	GCCCATCTGTAAGCGATAG	16S	74	60.5	52.6%	28.5	20.2
BH2-Probe	FAM-CCACGCTTTTGAAAGATGGTTT-BHQ1			69.5	40.9%		
BH3-Fwd	TAGGGAATCGCACGAATGGAA			65.5	47.6%		
BH3-Rev	ATGCTTCGCGAGAAGCG	23S	77	62	52.6%	30.8	22.0
BH3-Probe	FAM-TGCGTCCAAGCAGTGAGTGTGAG-BHQ1			76.6	56.5%		
BH4-Fwd	AGTGCTAATTGTTTAACCG			55.4	36.8%		
BH4-Rev	CGCACATTTCCATTCGT	23S	66	59.5	47.1%	33.7	16.4
BH4-Probe	FAM-TGGGGTGACACAGAAGGATA-BHQ1			66.9	50.0%		
BH5-Fwd	GATAAGAGTAACTGCTTGTC			57.3	40.0%		
BH5-Rev	CTGCTGGCACGTAGTTA	16S	68	60.9	52.9%	28.9	18.4
BH5-Probe	FAM-CTTGACGGTATCTAACCAGA-BHQ1			67.1	45.0%		

^a^FAM, 6-carboxyfluorescein; BHQ1: Black Hole Quencher 1; Probe with a Black Hole Quencher® modification at the 3' end (LGC, Biosearch Technologies, Petaluma, CA)

^b^Annealing temperatures were determined by either using Geneious 6.1.8 software (Biomatters, Inc., San Diego, CA) for the LR oligo set (Kearse et al. 2012) or RealTimeDesign™ software (LGC, Biosearch Technologies) for the BH oligo sets (Sowers et al. 2005, 2006)

^c^As described in the Materials and Methods, cycle threshold (Ct) values were obtained from RT-qPCR amplifications of *Listeria monocytogenes* and *Bacillus cereus* RNA using the TaqPath™ 1-Step Multiplex Master Mix (Applied Biosystems, Foster City, CA) in a QuantStudio™ 5 Real-Time PCR System (Applied Biosystems) with the following parameters: RT step at 54 °C for 15 min, hot start at 95 °C for 2 min followed by 40 cycles each of denaturation at 95 °C for 3 s and annealing and extension at 64 °C for 30 s. Ct values were analyzed using the QuantStudio™ Design & Analysis software (Applied Biosystems)

### Assay specificity for target RNA detection in *Listeria* species

As a preliminary screening test, the various candidates of designed oligonucleotides sets were subjected to an initial inclusivity test using *L. monocytogenes* as a representative targeted species, and an exclusivity test was conducted using *B. cereus* as a representative non-targeted species, which was based on previous whole-genome comparisons suggesting that high conservation in the genome organization is specific for *Bacillus* and *Listeria* (Buchrieser et al. 2003). Amplifications for detecting *L. monocytogenes* with 10 fg of RNA as template, corresponding to 10 cell equivalents (Milner et al. 2001), resulted in Ct values, ranging from 28.5 to 33.7 for all of the oligonucleotide sets (Table 2). However, only the BH1 oligonucleotide set was found to show no cross reactivity for detecting *B. cereus* in the exclusivity test even at template amounts that were 100-fold when compared to the targeted *Listeria* species. The other candidate oligonucleotide sets showed a positive reaction resulting in Ct values ranging from 16.4 to 32.5 (Table 2). Based on these initial observations determining the specificity of the amplification reaction, the subsequent validation tests employed the BH1 oligonucleotide set.

**Fig. 2:**
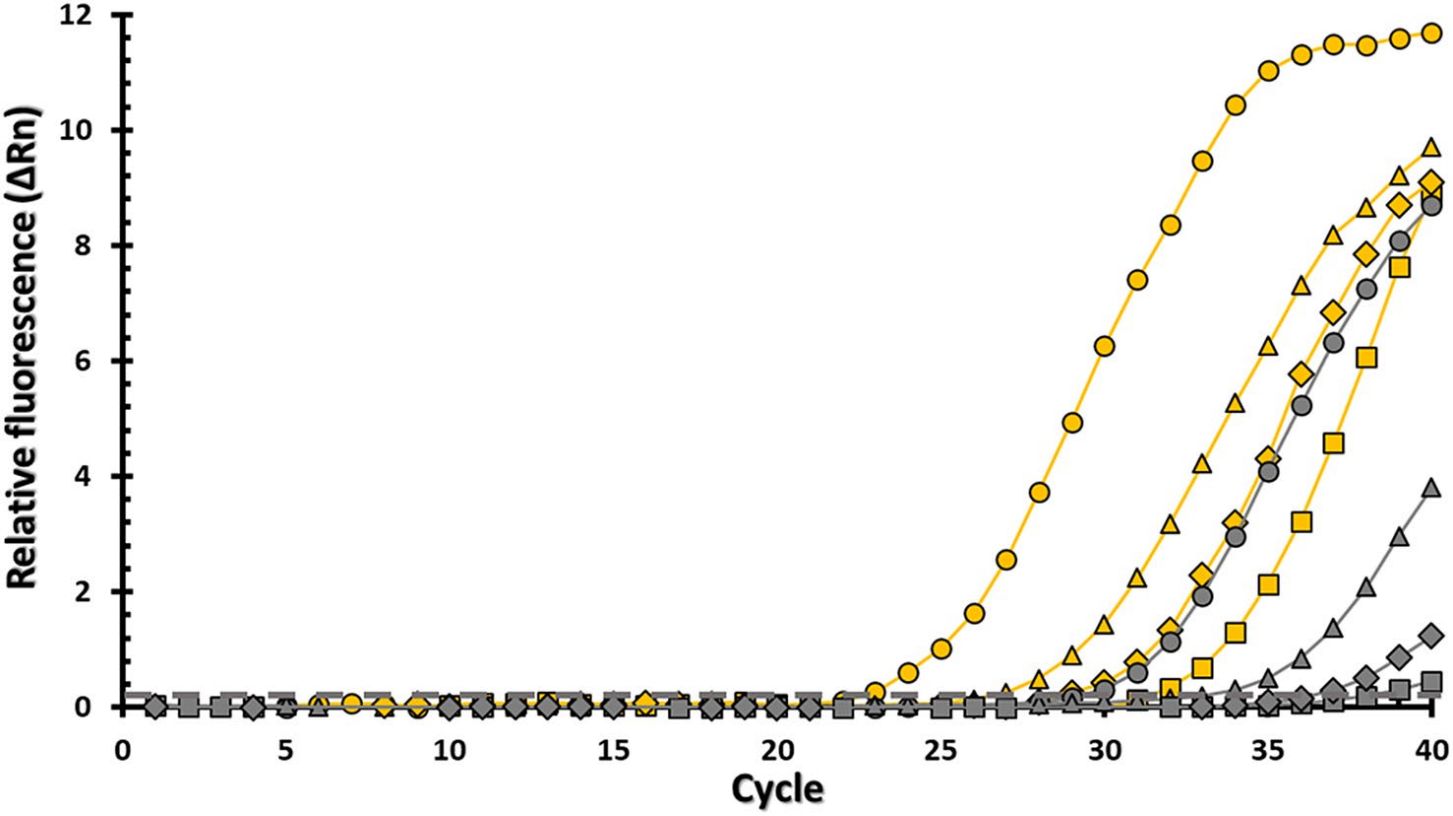
Improved sensitivity of the RNA-based assay when compared to a DNA-based assay. The Y-axis shows the relative fluorescence (ΔRn), the X-axis shows the number of amplification cycles. Yellow symbols represent data obtained with the RNA-based assay, and gray symbols represent data obtained with the DNA-based assay, MicroSEQ™ *Listeria monocytogenes* Detection kit (Applied Biosystems). Representative data are shown for approximately 5,000 bacterial cells (circles), 500 bacterial cells (triangles), 50 bacterial cells (diamonds), and 5 bacterial cells (squares). The detection threshold is indicated by the dashed line

The performance of the BH1 oligonucleotide-based assay, targeting multicopy sequences in the rRNA, was further compared to a DNA-based assay, which targets a single copy of an essential gene (*rnpB*) in *Listeria* species (Mandin et al. 2007; Petrauskene et al. 2017; Yusuf et al. 2010) (Fig. 2). In particular, cell lysates of *L. monocytogenes* outbreak strain RM2199 were prepared at various tenfold dilutions, ranging from 5000 to 5 cell equivalents (Fig. 2). Aliquots of the cell lysate were subjected to amplification by performing RT-qPCR using the BH1 oligonucleotide-based assay or just PCR using the DNA-based assay targeting *rnpB*. As shown in Fig. 2, the RNA-based assay with the BH1 probe set resulted in Ct values of 22.7 and 26.9 at the 5000 and 500 cell equivalents, respectively. By contrast, the DNA-based assay resulted in Ct values of 29.1 and 32.1 at the 5000 and 500 cell equivalents, respectively. Interestingly, the BH1 oligonucleotide-based assay still resulted in Ct values of 29.1 and 31.6 at the 50 and 5 cell equivalents, respectively; however, at these lower amounts of *Listeria* cell equivalents, the DNA-based assay yielded no detectable or Ct values > 35, considered to be a threshold detection limit for gene amplification (McCall et al. 2014). When compared to the DNA-based assay tested, the RNA-based assay, using the BH1 oligonucleotide set, reliably detected cell amounts lower than 5 cells, and these detection sensitivities were statistically significant (Fisher’s Exact Test, *P*-value = 0.002). These findings revealed that targeting multicopy RNA sequences significantly improved the detection capabilities of the assay for *L. monocytogenes*, and the results showed that the BH1 oligonucleotide-based assay was found to be at least 100 times more sensitive than the DNA-based assay targeting a single copy gene.

**Fig. 3:**
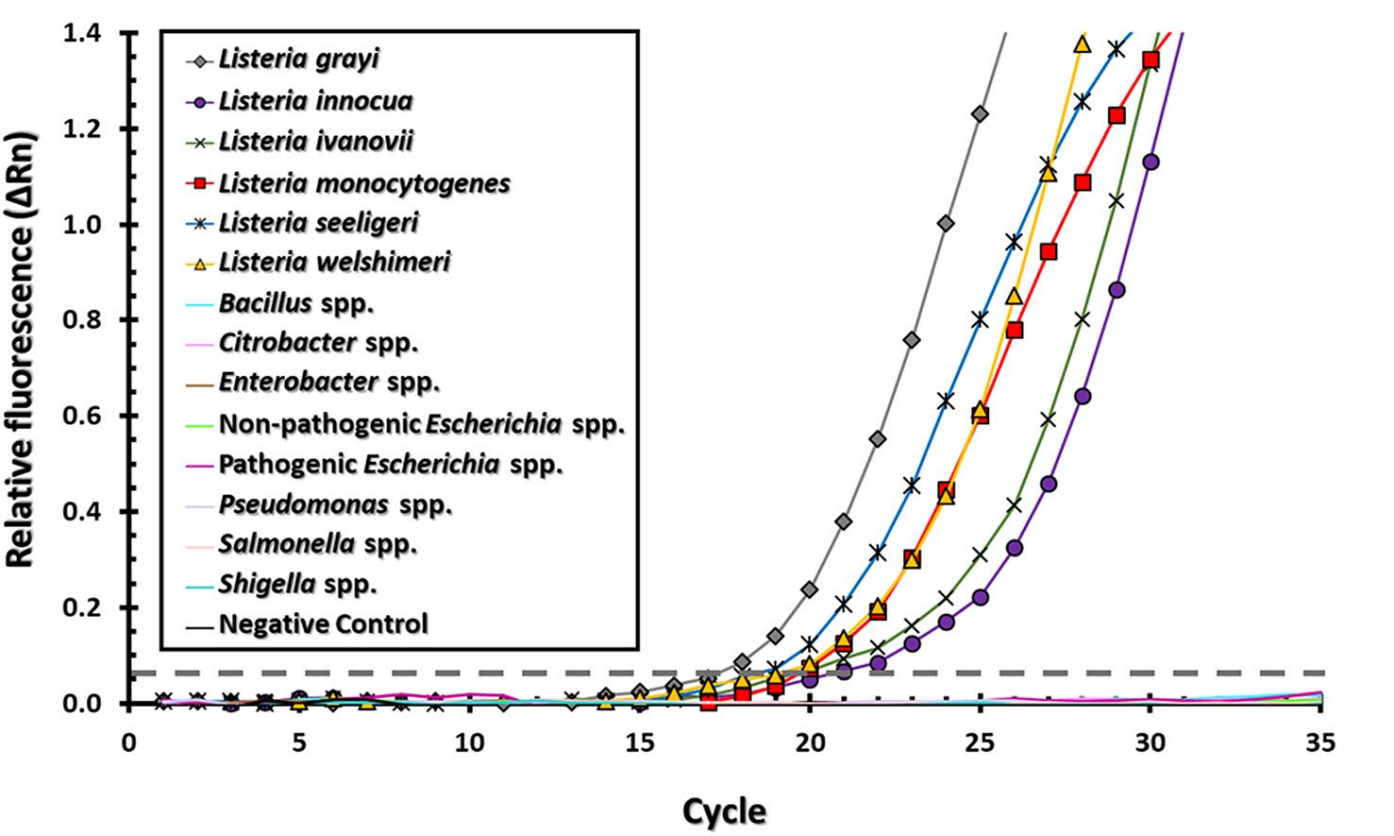
Specificity of the oligonucleotide-based assay for target RNA detection. The Y-axis shows the relative fluorescence (ΔRn), the X-axis shows the number of amplification cycles. Representative data are shown for strains of *L. grayi* (diamonds), *L. innocua* (circles), *L. ivanovii* (crosses), *L. monocytogenes* (squares), *L. seeligeri* (asterisks), and *L. welshimeri* (triangles). Data obtained for the negative control and for other gram-positive or gram-negative non-target bacterial strains are presented with lines without symbols. The detection threshold is indicated by the dashed line

To further validate the detection capabilities of the assay, the specificity of the BH1 oligonucleotide set was further expanded by testing for *Listeria* species other than *L. monocytogenes*. Other additional tested species were *L. innocua, L. ivanovii, L. welshimeri,* and *L. seeligeri*, which have been potentially implicated in causing illness to immunocompromised individuals (Korsak and Szuplewska 2016) and have been recovered from ready-to-eat foods or raw/unprocessed foods (Arslan and Özdemir 2020; Guerra et al. 2001; Pesavento et al. 2010; Soriano et al. 2001; Zeinali et al. 2017) as well as in food processing facilities (Huang et al. 2007; Korsak and Szuplewska 2016), and food products (Chen et al. 2009; Pesavento et al. 2010). The inclusivity test was conducted using RNA (100 pg) from *Listeria* species*,* corresponding to an estimated 100–150 cell equivalents of RNA per amplification reaction (Glaser et al. 2001; Milner et al. 2001) (Fig. 3). These experiments also examined for cross hybridization of the BH1 oligonucleotide set with excess of non-target RNA (20 ng), which is equivalent to approximately 2 million non-target cells and 4 billion copies of non-target RNA sequences, using an estimated million cell equivalent of RNA per test (Milner et al. 2001) from environmental bacterial species from soil, water, and plant surfaces (Dees et al. 2015; Williams and Marco 2014; Williams et al. 2013) or other foodborne pathogens (Fig. 3). The results indicated a high level of specificity for detecting various *Listeria* species with Ct values ranging from 19.9 to 25.0, showing a statistically significant difference (Fisher’s Exact Test, *P*-value = 0.0006) when compared to the lack of detected signals for non-targeted environmental and pathogenic bacterial strains.

**Fig. 4:**
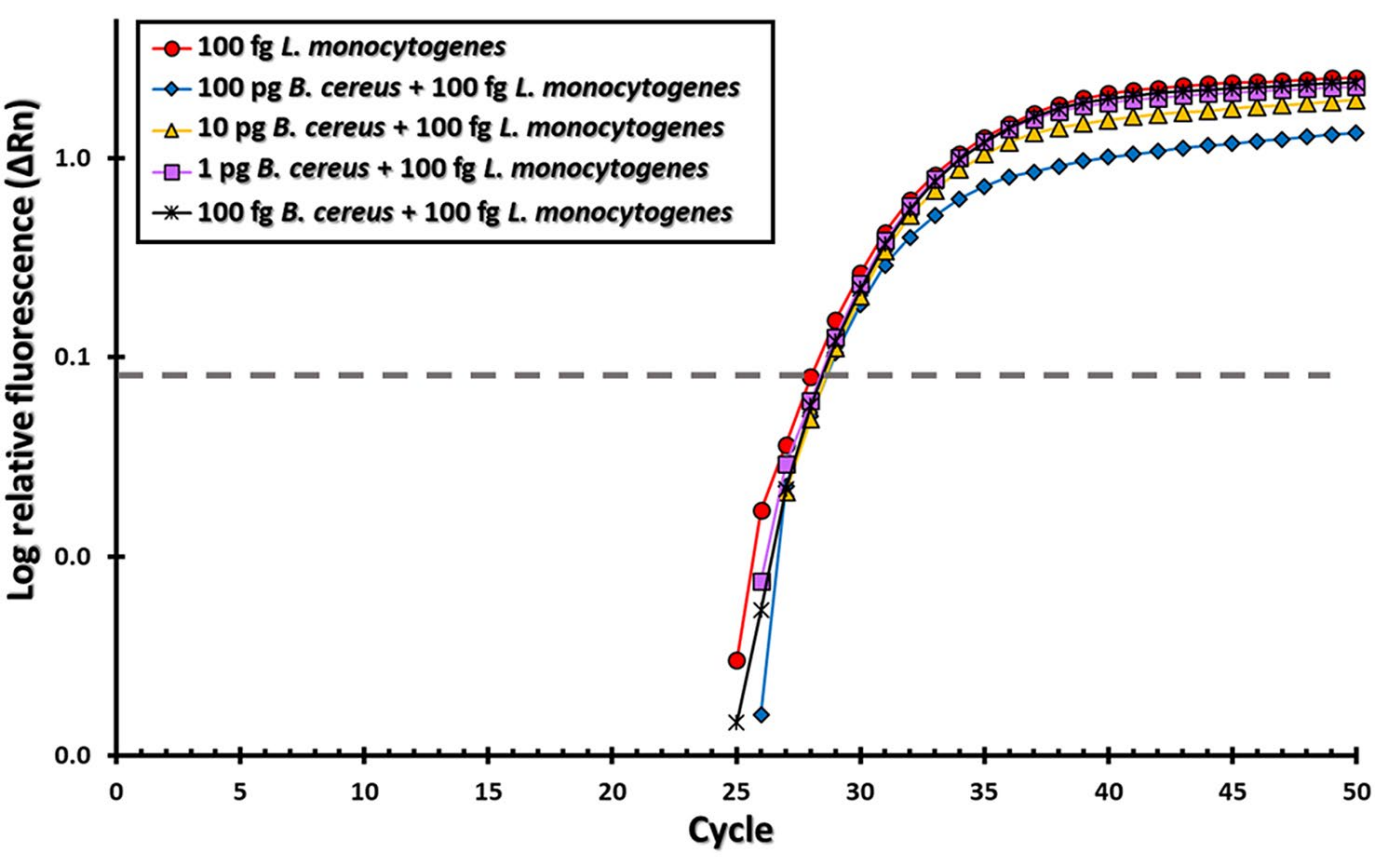
Co-incubation of target RNA from *L. monocytogenes* strain RM2199 in the presence of excess non-target RNA from *B. cereus strain* ATCC 14,579. The Y-axis shows the relative fluorescence (ΔRn), the X-axis shows the number of amplification cycles. Representative data are shown for 100 fg *L. monocytogenes* (circles), 100 fg *L. monocytogenes* plus 100 pg *B. cereus* (diamonds), 100 fg *L. monocytogenes* plus 10 pg *B. cereus* (triangles), 100 fg *L. monocytogenes* plus 1 pg *B. cereus* (squares), and asterisks represent data for 100 fg *L. monocytogenes* plus 100 fg *B. cereus* (asterisks). The detection threshold is indicated by a dashed line

Based on recent evidence indicating that the residential bacterial communities are able to persist over time on food production surfaces (Møretrø and Langsrud 2017), additional experiments examined the effect of various amounts of non-target RNA on the efficiency of the amplification of the *Listeria* sequences. Purified target RNA from *L. monocytogenes* was co-incubated in the presence of various amounts of excess non-target RNA from *B. cereus*, a persistent species in food industry environments (Møretrø and Langsrud 2017) and displaying a sequence similarity greater than 90% in the 16S ribosome region (Sallen et al. 1996). As shown in Fig. 4, the results indicated that no significant differences in the Ct values were observed with values ranging from 27.8 to 28.3, corresponding to the signal detected for *L. monocytogenes* strain RM2199 in the presence of excess non-target RNA from *B. cereus* strain ATCC 14,579. To determine the effect of the various tested conditions on the amplification efficiency of the target, the slope of the curve at the pre-inflection point was examined (Guescini et al. 2013). Analysis of the amplification curve resulted in no significant change in the slope of the curve under the various conditions tested, and a one-way ANOVA test indicated that the differences between the tested conditions, varying the amounts of non-target template, were not statistically significant (*df* = 4, *F* = 3.266, *F* critical = 5.192). These observations demonstrated that the efficiency of the amplification specific for *Listeria* was not adversely affected by addition of the non-target template.

### Design and validation of a continuous flow-through system for *Listeria* species detection

**Fig. 5:**
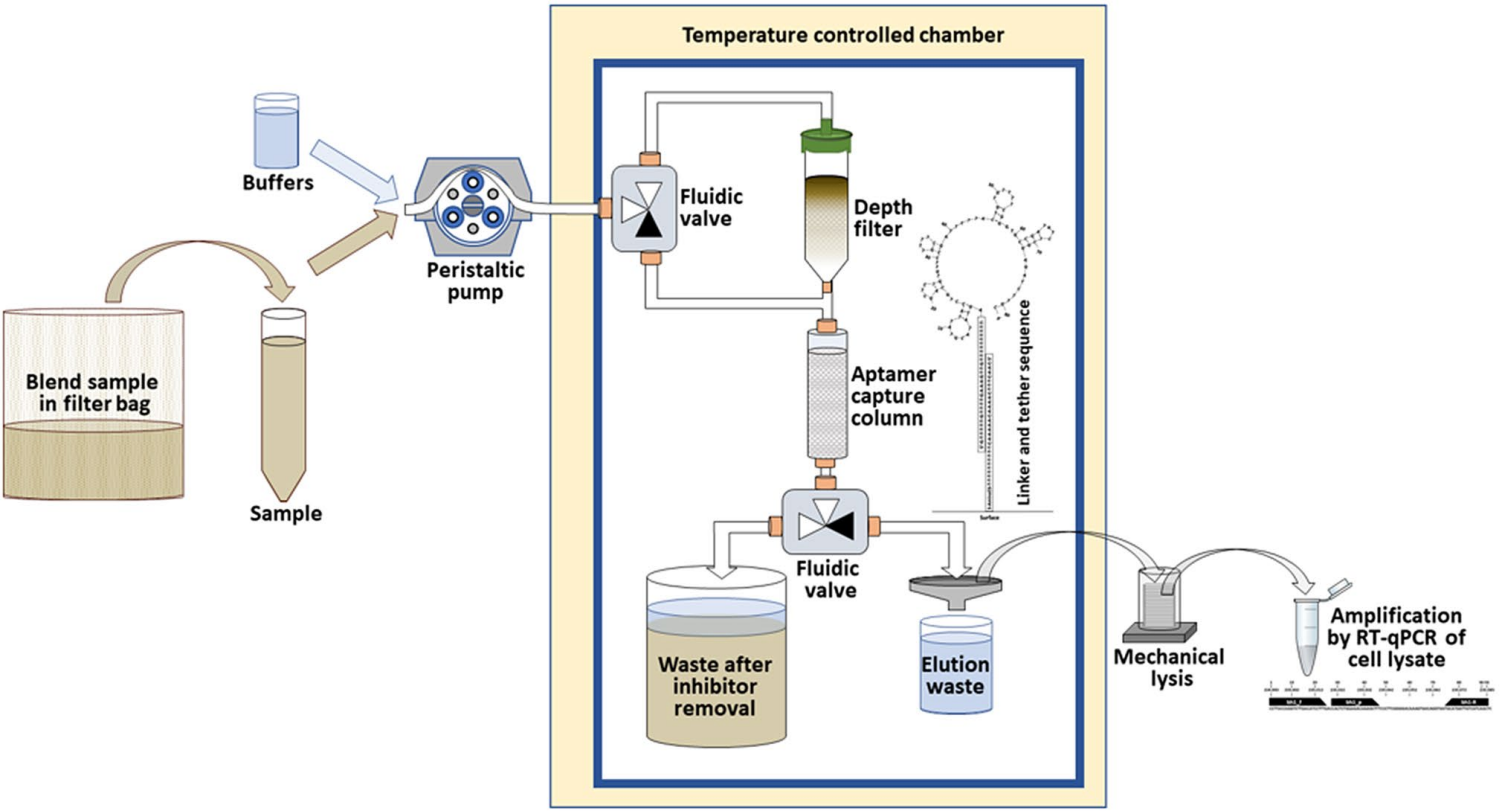
Schematic diagram of the sample processing steps in the continuous flow-through system. Sample was blended with capture buffer, filtered to remove large particulates, and introduced into a temperature-controlled chamber using a peristaltic pump. Fluidic valves moved homogenized sample to a depth filter, and *Listeria* cells in the sample were captured with an aptamer-functionalized column. Potential inhibitors were removed as waste, and the cells were subjected to mechanical lysis. The lysed cells were collected and subjected to further amplification by RT-qPCR

To incorporate the use of the BH1 oligonucleotide-based assay with an improved detection platform, a flow-through system was designed for the real-time surveillance of *Listeria* species in food processing facilities (Medin et al. 2020). By avoiding the need for sample preparation processes utilizing multistage centrifugation to concentrate the targeted pathogen, the present study replaced the input centrifugation with a flow-through system that included a depth filter to remove large particulate matter while permitting target pathogen to pass through a DNA aptamer-functionalized capture column that specifically bound *Listeria* cells (Fig. 5). In detail, the filtered sample was introduced into a temperature-controlled chamber (10 °C–12 °C), and fluidic valves moved homogenized sample to a depth filter, which removed large particulate matter present in the collected sample, while still permitting target pathogen to pass through to a microbead capture column that specifically bound *Listeria* cells (Fig. 5). The specific binding in the capture column was achieved by the use of DNA aptamers (Suh et al. 2014; Suh and Jaykus 2013), which are oligonucleotide molecules that bind to specific epitopes that are presented on the bacterial cell surface and have been proposed for pathogen capture as a low-cost alternative to antibodies (Teng et al. 2016). The use of DNA aptamers enabled *Listeria* cell capturing without the need for small particle filtering, which can be subject to membrane fouling when isolating bacterial pathogens from food and environmental samples (Ferrari et al. 2019; Kearns et al. 2019; Li et al. 2013; Zhang et al. 2018). To improve the capturing of the *Listeria* cells with the DNA aptamer (Suh et al. 2014; Suh and Jaykus 2013), additional sequences, a spacer sequence for aptamer extension and a tether sequence for aptamer surface attachment (see Material and Methods)(Quiñones et al. 2020), were designed and added to the aptamer to enable binding to the capture column and prevent the aggregation of the aptamer for efficient release of the target bacterial cell from the aptamer (Medin et al. 2020; Quiñones et al. 2020). A fluidic valve was used to bypass the depth filter, and potential inhibitors were removed as waste. The captured *Listeria* cells were released from the aptamer-functionalized column, and the liquid sample volume with the *Listeria* cells was reduced and further subjected to mechanical lysis and amplification with the BH1 oligonucleotide-based assay (Fig. 5). In summary, the designed flow-through system included a sample preparation process with DNA aptamers to specifically capture the targeted *Listeria* cells away from the contaminating matrix and other non-targeted organisms and inhibitors prior to nucleic acid amplification and analysis.

**Fig. 6:**
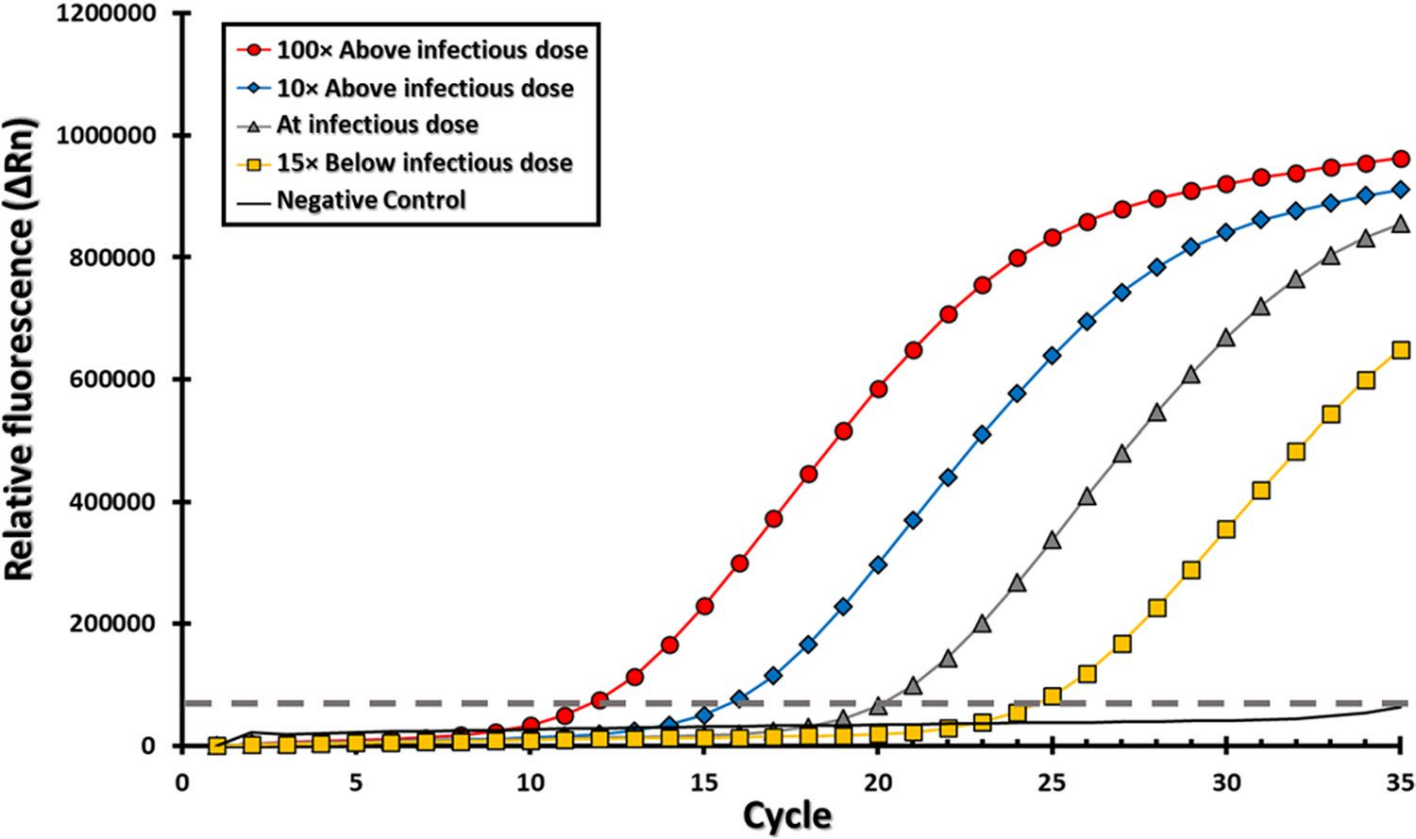
Detection sensitivity of the oligonucleotide-based assay with the continuous flow-through system. The Y-axis shows the relative fluorescence (ΔRn), the X-axis shows the number of amplification cycles. Representative data are shown for samples containing *L. grayi* strain RM2208 at equivalent amounts of 100 times above the *Listeria* infectious dose (circles), at 10 times above the *Listeria* infectious dose (diamonds), at the *Listeria* infectious dose (triangles), at 15 times below the *Listeria* infectious dose (squares). The negative control is indicated by the gray line. The detection threshold is indicated by the dashed line

As a proof-of-concept, the ability of the flow-through system for efficiently capturing and detecting *Listeria* cells was further examined. To improve the capture binding efficiency of the *Listeria* cells, the capture time in the flow-through system was optimized by considering the function of the cross-sectional area of the flow channel and the surface area of the capture surface (Medin et al. 2020). In addition, the aptamer-capture column employed a functionalized surface chemistry based on soda-lime glass surfaces (Medin et al. 2020), one of the least expensive and most-commonly used glass types for many applications. By flowing cell suspensions of *L. grayi*, a non-pathogenic *Listeria* species found in raw and ready-to-eat foods (Orsi and Wiedmann 2016; Soriano et al. 2001), through the capture column, the results indicated a 78% capture efficiency of the targeted *L. grayi* strain RM2208. Given that the tested DNA aptamer may display some affinity for closely related species (Suh et al. 2014; Suh and Jaykus 2013; Teng et al. 2016), *Listeria* cells captured via aptamer were differentiated using the BH1 oligonucleotide set. To further validate the detection sensitivities of the flow-through system, *L. grayi* strain RM2208 cell suspensions, consisting of various amounts at or below the infectious dose, based on an estimated infectious dose of *Listeria* at about 10,000 cells (Pouillot et al. 2016), were determined by traditional plate enumeration on selective solid media. After elution from the capture and ultrasonic mechanical lysis, the results revealed Ct values of 12.3, 16.3, 20.9, and 24.9 for *L. grayi* cell amounts that were 100 times above the infectious dose, 10 times above the infectious dose, at the infectious dose, and 15 times below the infectious dose, respectively (Fig. 6). Further analysis of the Ct values using the tested serial dilutions of the *Listeria* cell suspensions were used to calculate the efficiency of the amplification (Kubista et al. 2006), and the analysis revealed that the efficiency of the amplification for detecting *Listeria* with the flow-through system in conjunction with the BH1 oligonucleotide set had an estimated amplification efficiency of 0.9235 (*R* = 0.99955; slope -3.52). These findings indicated that this integrated flow-through system has the capabilities to accurately and sensitively detect *Listeria* species.

## Conclusions

In conclusion, the present study developed a singleplex RT-qPCR assay using the BH1 oligonucleotide set for the accurate detection of multiple *Listeria* species. Previously published assays for *Listeria* species detection employed qPCR and were developed by targeting *iap*, *plcB*, and *scrA* genes, required for virulence and regulatory functions, as well as putative internalin and oxidoreductase, and N-acetylmuramidase proteins (Chen et al. 2017; Hage et al. 2014; Hein et al. 2001; Hitchins et al. 2017). Other qPCR assays have been documented, which target the 23S ribosomal DNA or non-coding RNA specific to the *Listeria* genus (Chen et al. 2017; Petrauskene et al. 2017; Rodríguez-Lázaro et al. 2004). The disadvantage of these published assays is the requirement of multiple set of primers and probes to be multiplexed per amplification reaction to enable the detection of various *Listeria* species.

As an alternative to DNA-based methods by employing qPCR, the present study developed a RT-qPCR assay that detected rRNA in *Listeria* species. The advantage of using rRNA as the target in *Listeria* species identification is that the rRNA genes are present in this pathogen at all stages of growth, accumulate mutations at a slow constant rate, and contain sequences that enable discrimination of this targeted pathogen at the genus level (Espejo and Plaza 2018; Milner et al. 2001). Additionally, analysis of the *Listeria* genome revealed large amounts of rRNA (600–25,000 copies per cell) (Milner et al. 2001), which makes rRNA a better target for a sensitive detection at low cell concentrations when compared with one copy of genomic DNA for a targeted gene. The explosive use of genome sequencing has resulted in an increase in the growth of data (Land et al. 2015; Sayers et al. 2020), which has consequently improved the in silico sequence analyses for the identification of rRNA sequences that are highly specific for the targeted pathogen. In the present study, the designed singleplex RT-qPCR assay using the BH1 oligonucleotide assay, targeting 23S rRNA, resulted in the reliable detection of less than 5 *Listeria* cells, and this detection was at least 100 times more sensitive than the DNA-based assay targeting a single copy gene. Furthermore, the BH1 oligonucleotide assay results showed no cross reactivity for detecting non-*Listeria* strains in the exclusivity test even at template amounts that were 100-fold when compared to the targeted *Listeria* species.

The use of enrichment in routine surveillance testing is traditionally performed to amplify the target organism exponentially by as much as a millionfold so that detection is possible (Law et al. 2015). To improve the detection of pathogens from samples, various reports have documented on using microfiltration to reduce large samples to a small volume (Li et al. 2013). However, small particle filtering can be subject to membrane fouling when isolating bacterial pathogens from food and environmental samples, resulting in reduced levels of detection sensitivities (Kearns et al. 2019; Li et al. 2013; Zhang et al. 2018). In the present study, the flow-through system did not use membrane filtration but instead used an adaptation of depth filtering, a processing stage previously used for preventing filter clogging (Murakami 2012), followed by the use of an aptamer-functionalized column for successfully capturing and concentrating the targeted *Listeria* cells from samples. The findings from the present study indicated the flow-through system accurately detected *Listeria* cells at cell concentrations that were 15 times below the infectious dose. Due to the non-uniform distribution of microorganisms, current methods employing small sample sizes can result in undetected pathogens, and the lack of accurate detection of the tested foodborne pathogen can lead to subsequent outbreaks (Capobianco et al. 2021; Kearns et al. 2019). To continue to meet the needs of the food safety industry and regulators (Kaplan et al. 2014; Kuiper and Paoletti 2015), further research is aimed at optimizing the high binding efficiency of the flow-through system, documented in the present study, to optimize the rapid, low-cost capture of *Listeria* species using increased sample size over a range of environmental and food samples. Moreover, recent preliminary evidence demonstrated that the flow-through system accurately detected *Listeria* at low cell concentrations, ranging from 3.5 CFU/ml to 1000 CFU/ml, in samples collected at a food processing facility (Quiñones et al. 2019, 2020) and these challenging samples were characterized by containing contaminants commonly found in soils and agricultural products, including debris and humic acids, which are known inhibitors of nucleic acid amplification (Law et al. 2015; Opel et al. 2010; Schrader et al. 2012). These observations indicate that the high capture efficiency and strong binding affinity of aptamers towards the targeted *Listeria* cells enable aggressive washes to remove inhibitors and substances that negatively impact nucleic acid amplification and would thus enable the detection of *Listeria* cells in other types of samples such as complex food matrices, known to contain PCR inhibitory substances (Schrader et al. 2012). Future studies will also explore the increased sampling size of the method, described in the present study, to improve the statistical significance of the testing procedure and to enable a sampling process that is more accurate and representative of the entire agricultural field, providing an added value to the food industry.

## Data Availability

The material analyzed during the current study is available from the corresponding author on reasonable request.

## References

[CR1] Arslan S, Özdemir F (2020). Prevalence and antimicrobial resistance of *Listeria* species and molecular characterization of *Listeria monocytogenes* isolated from retail ready-to-eat foods. FEMS Microbiol Lett.

[CR2] Bettelheim KA, Evangelidis H, Pearce JL, Sowers E, Strockbine NA (1993). Isolation of a *Citrobacter freundii* strain which carries the *Escherichia coli* O157 antigen. J Clin Microbiol.

[CR3] Brandl MT, Mandrell RE (2002). Fitness of *Salmonella enterica* serovar Thompson in the cilantro phyllosphere. J Appl Environ Microbiol.

[CR4] Brouillette R, Aggen D, Borchert B, Buckman K, Domanico M, Fraser-Heaps J, Freier T, Hayman M, Jackson T, Kataoka A, Meyer J, Shoaf E, Stone W (2014). *Listeria monocytogenes* guidance on environmental monitoring and corrective actions in at-risk foods.

[CR5] Buchanan RL, Gorris LGM, Hayman MM, Jackson TC, Whiting RC (2017). A review of *Listeria monocytogenes*: An update on outbreaks, virulence, dose-response, ecology, and risk assessments. Food Control.

[CR6] Buchrieser C, Rusniok C, Kunst F, Cossart P, Glaser P, Frangeul L, Amend A, Baquero F, Berche P, Bloecker H, Brandt P, Chakaborty T, Charbit A, Chétouani F, Couvé E, De Daruvar A, Dehoux P, Domann E, Domínguez-Bernal G, Duchaud E, Durand L, Dusurget O, Entian KD, Fsihi H, Garcia-Del Portillo P, Garrido P, Gautier L, Goebel W, Gómez-López N, Hain T, Hauf J, Jackson D, Jones LM, Kärst U, Kreft J, Kuhn M, Kurapkat G, Madueño E, Maitournam A, Mata Vicente J, Ng E, Nordsiek G, De Pablos B, Pérez-Diaz JC, Remmel B, Rose M, Schlueter T, Simoes N, Vázquez-Boland JA, Voss H, Wehland J (2003). Comparison of the genome sequences of *Listeria monocytogenes* and *Listeria innocua*: Clues for evolution and pathogenicity. FEMS Immunol Med Microbiol.

[CR7] Capobianco JA, Armstrong CM, Lee J, Gehring AG (2021). Detection of pathogenic bacteria in large volume food samples using an enzyme-linked immunoelectrochemical biosensor. Food Control.

[CR8] Charlermroj R, Makornwattana M, Phuengwas S, Meerak J, Pichpol D, Karoonuthaisiri N (2019). DNA-based bead array technology for simultaneous identification of eleven foodborne pathogens in chicken meat. Food Control.

[CR9] Chen J, Zhang X, Mei L, Jiang L, Fang W (2009). Prevalence of *Listeria* in Chinese food products from 13 provinces between 2000 and 2007 and virulence characterization of *Listeria monocytogenes* isolates. Foodborne Pathog Dis.

[CR10] Chen JQ, Healey S, Regan P, Laksanalamai P, Hu Z (2017). PCR-based methodologies for detection and characterization of *Listeria monocytogenes* and *Listeria ivanovii* in foods and environmental sources. Food Sci Hum Well.

[CR11] Churchill KJ, Sargeant JM, Farber JM, O’connor AM, (2019). Prevalence of *Listeria monocytogenes* in select ready-to-eat foods—deli meat, soft cheese, and packaged salad: A systematic review and meta-analysis. J Food Prot.

[CR12] Cooley MB, Miller WG, Mandrell RE (2003). Colonization of *Arabidopsis thaliana* with *Salmonella enterica* and enterohemorrhagic *Escherichia col*i O157:H7 and competition by *Enterobacter asburiae*. J Appl Environ Microbiol.

[CR13] Cooley M, Carychao D, Crawford-Miksza L, Jay MT, Myers C, Rose C, Keys C, Farrar J, Mandrell RE (2007). Incidence and tracking of *Escherichia coli* O157:H7 in a major produce production region in California. PLoS ONE.

[CR14] Cooley MB, Jay-Russell M, Atwill ER, Carychao D, Nguyen K, Quiñones B, Patel R, Walker S, Swimley M, Pierre-Jerome E, Gordus AG, Mandrell RE (2013). Development of a robust method for isolation of Shiga toxin-positive *Escherichia coli* (STEC) from fecal, plant, soil and water samples from a leafy greens production region in California. PLoS ONE.

[CR15] Cooley MB, Quiñones B, Oryang D, Mandrell RE, Gorski L (2014). Prevalence of Shiga toxin producing *Escherichia coli*, *Salmonella enterica*, and *Listeria monocytogenes* at public access watershed sites in a California Central Coast agricultural region. Front Cell Infect Microbiol.

[CR16] Dees MW, Lysøe E, Nordskog B, Brurberg MB (2015). Bacterial communities associated with surfaces of leafy greens: Shift in composition and decrease in richness over time. J Appl Environ Microbiol.

[CR17] Doumith M, Buchrieser C, Glaser P, Jacquet C, Martin P (2004). Differentiation of the major *Listeria monocytogenes* serovars by multiplex PCR. J Clin Microbiol.

[CR18] Espejo RT, Plaza N (2018). Multiple ribosomal RNA operons in bacteria; Their concerted evolution and potential consequences on the rate of evolution of their 16S rRNA. Front Microbiol.

[CR19] Estrada EM, Hamilton AM, Sullivan GB, Wiedmann M, Critzer FJ, Strawn LK (2020). Prevalence, persistence, and diversity of *Listeria monocytogenes* and *Listeria* species in produce packinghouses in three U.S. States J Food Prot.

[CR20] Fagerquist CK, Zaragoza WJ (2018). Proteolytic surface-shaving and serotype-dependent expression of SPI-1 invasion proteins in *Salmonella enterica s*ubspecies *enterica*. Front Nutr.

[CR21] Feil H, Feil WS, Chain P, Larimer F, DiBartolo G, Copeland A, Lykidis A, Trong S, Nolan M, Goltsman E, Thiel J, Malfatti S, Loper JE, Lapidus A, Detter JC, Land M, Richardson PM, Kyrpides NC, Ivanova N, Lindow SE (2005). Comparison of the complete genome sequences of *Pseudomonas syringae* pv. *syringae* B728a and pv. tomato DC3000. Proc Natl Acad Sci U S A.

[CR22] Ferrari S, Frosth S, Svensson L, Fernström LL, Skarin H, Hansson I (2019). Detection of *Campylobacter* spp. in water by dead-end ultrafiltration and application at farm level. J Appl Microbiol.

[CR23] Fisher RA (1935). The Logic of Inductive Inference. J R Stat Soc.

[CR24] Friedman M, Henika PR, Levin CE, Mandrell RE, Kozukue N (2006). Antimicrobial activities of tea catechins and theaflavins and tea extracts against *Bacillus cereus*. J Food Prot.

[CR25] Gasanov U, Hughes D, Hansbro PM (2005). Methods for the isolation and identification of *Listeria* spp. and *Listeria monocytogenes*: A review. FEMS Microbiol Rev.

[CR26] Glaser P, Frangeul L, Buchrieser C, Rusniok C, Amend A, Baquero F, Berche P, Bloecker H, Brandt P, Chakraborty T, Charbit A, Chetouani F, Couvé E, De Daruvar A, Dehoux P, Domann E, Domínguez-Bernal G, Duchaud E, Durant L, Dussurget O, Entian KD, Fsihi H, Garcia-Del Portillo F, Garrido P, Gautier L, Goebel W, Gómez-López N, Hain T, Hauf J, Jackson D, Jones LM, Kaerst U, Kreft J, Kuhn M, Kunst F, Kurapkat G, Madueño E, Maitournam A, Mata Vicente J, Ng E, Nedjari H, Nordsiek G, Novella S, De Pablos B, Pérez-Diaz JC, Purcell R, Remmel B, Rose M, Schlueter T, Simoes N, Tierrez A, Vázquez-Boland JA, Voss H, Wehland J, Cossart P (2001). Comparative genomics of *Listeria* species. Science.

[CR27] Gorski L, Flaherty D, Mandrell RE (2006). Competitive fitness of *Listeria monocytogenes* serotype 1/2a and 4b strains in mixed cultures with and without food in the U.S. Food and Drug Administration enrichment protocol. J Appl Environ Microbiol.

[CR28] Guerra MM, McLauchlin J, Bernardo FA (2001). *Listeria* in ready-to-eat and unprocessed foods produced in Portugal. Food Microbiol.

[CR29] Guescini M, Sisti D, Rocchi MBL, Panebianco R, Tibollo P, Stocchi V (2013). Accurate and precise DNA quantification in the presence of different amplification efficiencies using an improved *Cy0* method. PLoS ONE.

[CR30] Hage E, Mpamugo O, Ohai C, Sapkota S, Swift C, Wooldridge D, Amar CFL (2014). Identification of six *Listeria* species by real-time PCR assay. Lett Appl Microbiol.

[CR31] Hein I, Klein D, Lehner A, Bubert A, Brandl E, Wagner M (2001). Detection and quantification of the *iap* gene of *Listeria monocytogenes* and *Listeria innocua* by a new real-time quantitative PCR assay. Res Microbiol.

[CR32] Hitchins AD, Jinneman K, Chen Y (2017) BAM Chapter 10: Detection of *Listeria monocytogenes* in foods and environmental samples, and enumeration of *Listeria monocytogenes* in foods. https://www.fda.gov/food/laboratory-methods-food/bam-detection-and-enumeration-listeria-monocytogenes. Accessed 03/04/2020

[CR33] Hoffmann S, Scallan Walter E (2019). Acute complications and sequelae from foodborne infections: Informing priorities for cost of foodborne illness estimates. Foodborne Pathog Dis.

[CR34] Hoffmann S, Batz MB, Morris JG (2012). Annual cost of illness and quality-adjusted life year losses in the United States due to 14 foodborne pathogens. J Food Prot.

[CR35] Hoffmann S, Maculloch B, Batz M (2015) Economic burden of major foodborne illnesses acquired in the United States. EIB-140, U.S. Department of Agriculture, Economic Research Service, May 2015. https://www.ers.usda.gov/webdocs/publications/43984/52807_eib140.pdf

[CR36] Huang B, Eglezos S, Heron BA, Smith H, Graham T, Bates J, Savill J (2007). Comparison of multiplex PCR with conventional biochemical methods for the identification of *Listeria* spp. isolates from food and clinical samples in Queensland. Australia J Food Prot.

[CR37] Ivanova N, Sorokin A, Anderson I, Galleron N, Candelon B, Kapatral V, Bhattacharyya A, Reznik G, Mikhailova N, Lapidus A, Chu L, Mazur M, Goltsman E, Larsen N, D'Souza M, Walunas T, Grechkin Y, Pusch G, Haselkorn R, Fonstein M, Ehrlich SD, Overbeek R, Kyrpides N (2003). Genome sequence of *Bacillus cereus* and comparative analysis with *Bacillus anthracis*. Nature.

[CR38] Kaplan RM, Chambers DA, Glasgow RE (2014). Big data and large sample size: A cautionary note on the potential for bias. J Clin Transl Sci.

[CR39] Kearns EA, Gustafson RE, Castillo SM, Alnughaymishi H, Lim DV, Ryser ET (2019). Rapid large-volume concentration for increased detection of *Escherichia coli* O157:H7 and *Listeria monocytogenes* in lettuce wash water generated at commercial facilities. Food Control.

[CR40] Kearse M, Moir R, Wilson A, Stones-Havas S, Cheung M, Sturrock S, Buxton S, Cooper A, Markowitz S, Duran C, Thierer T, Ashton B, Meintjes P, Drummond A (2012). Geneious Basic: An integrated and extendable desktop software platform for the organization and analysis of sequence data. Bioinformatics.

[CR41] Korsak D, Szuplewska M (2016). Characterization of nonpathogenic *Listeria* species isolated from food and food processing environment. Int J Food Microbiol.

[CR42] Kubista M, Andrade JM, Bengtsson M, Forootan A, Jonák J, Lind K, Sindelka R, Sjöback R, Sjögreen B, Strömbom L, Ståhlberg A, Zoric N (2006). The real-time polymerase chain reaction. Mol Aspects Med.

[CR43] Kuiper HA, Paoletti C (2015). Food and feed safety assessment: The importance of proper sampling. J AOAC Int.

[CR44] Kutyavin IV (2008). Use of base-modified duplex-stabilizing deoxynucleoside 5′-triphosphates to enhance the hybridization properties of primers and probes in detection polymerase chain reaction. Biochemistry.

[CR45] Kyle JL, Cummings CA, Parker CT, Quiñones B, Vatta P, Newton E, Huynh S, Swimley M, Degoricija L, Barker M, Fontanoz S, Nguyen K, Patel R, Fang R, Tebbs R, Petrauskene O, Furtado M, Mandrell RE (2012). *Escherichia coli* serotype O55:H7 diversity supports parallel acquisition of bacteriophage at Shiga toxin phage insertion sites during evolution of the O157:H7 lineage. J Bacteriol.

[CR46] Land M, Hauser L, Jun SR, Nookaew I, Leuze MR, Ahn TH, Karpinets T, Lund O, Kora G, Wassenaar T, Poudel S, Ussery DW (2015). Insights from 20 years of bacterial genome sequencing. Funct Integr Genomics.

[CR47] Law JW, Ab Mutalib NS, Chan KG, Lee LH (2015). Rapid methods for the detection of foodborne bacterial pathogens: Principles, applications, advantages and limitations. Front Microbiol.

[CR48] Li X, Ximenes E, Amalaradjou MAR, Vibbert HB, Foster K, Jones J, Liu X, Bhunia AK, Ladisch MR (2013). Rapid sample processing for detection of food-borne pathogens via cross-flow microfiltration. J Appl Environ Microbiol.

[CR49] Liu Y, Xu A, Fratamico PM, Sommers CH, Rotundo L, Boccia F, Jiang Y, Ward TJ (2018). Draft whole-genome sequences of seven *Listeria monocytogenes* strains with variations in virulence and stress responses. Microbiol Resour Announc.

[CR50] Livezey K, Kaplan S, Wisniewski M, Becker MM (2013). A new generation of food-borne pathogen detection based on ribosomal RNA. Annu Rev Food Sci Technol.

[CR51] Longhi C, Ammendolia MG, Conte MP, Seganti L, Iosi F, Superti F (2014). *Listeria ivanovii* ATCC 19119 strain behaviour is modulated by iron and acid stress. Food Microbiol.

[CR52] Mandin P, Geissmann T, Cossart P, Repoila F, Vergassola M (2007). Identification of new noncoding RNAs in *Listeria monocytogenes* and prediction of mRNA targets. Nucleic Acids Res.

[CR53] McCall MN, McMurray HR, Land H, Almudevar A (2014). On non-detects in qPCR data. Bioinformatics.

[CR54] Medin DL, De Guzman VS, Panchal Z (2020) System and method for detecting and monitoring pathogens. USA Patent United States Patent and Trademark Office, Patent Application Number 2020/043112

[CR55] Mehta CR, Patel NR (1986). Algorithm 643. FEXACT: a FORTRAN subroutine for Fisher's exact test on unordered *r*×*c* contingency tables. ACM Trans Math Softw..

[CR56] Milner MG, Saunders JR, McCarthy AJ (2001). Relationship between nucleic acid ratios and growth in *Listeria monocytogenes*. Microbiology.

[CR57] Møretrø T, Langsrud S (2017). Residential bacteria on surfaces in the food industry and their implications for food safety and quality. Compr Rev Food Sci Food Saf.

[CR58] Murakami T (2012). Filter-based pathogen enrichment technology for detection of multiple viable foodborne pathogens in 1 day. J Food Prot.

[CR59] Nielsen HB, Wernersson R, Knudsen S (2003). Design of oligonucleotides for microarrays and perspectives for design of multi-transcriptome arrays. Nucleic Acids Res.

[CR60] Niemira BA, Fan X, Sokorai KJB, Sommers CH (2003). Ionizing radiation sensitivity of *Listeria monocytogenes* ATCC 49594 and *Listeria innocua* ATCC 51742 inoculated on endive (*Cichorium endiva*). J Food Prot.

[CR61] Opel KL, Chung D, McCord BR (2010). A study of PCR inhibition mechanisms using real time PCR. J Forensic Sci.

[CR62] Orsi RH, Wiedmann M (2016). Characteristics and distribution of *Listeria* spp., including *Listeria* species newly described since 2009. Appl Microbiol Biotechnol.

[CR63] Pesavento G, Ducci B, Nieri D, Comodo N, Lo Nostro A (2010). Prevalence and antibiotic susceptibility of *Listeria* spp. isolated from raw meat and retail foods. Food Control.

[CR64] Petrauskene O, Cummings C, Vatta P, Tebbs R, Balachandran P, Zoder P, Wong L (2017) Detection of *Listeria* species in food and environmental samples, methods and compositions thereof. USA Patent United States Patent and Trademark Office, Patent Number 9,546,405 B2

[CR65] Pouillot R, Klontz KC, Chen Y, Burall LS, Macarisin D, Doyle M, Bally KM, Strain E, Datta AR, Hammack TS, Van Doren JM (2016). Infectious dose of *Listeria monocytogene*s in outbreak linked to ice cream, United States, 2015. Emerg Infect Dis.

[CR66] Quiñones B, Swimley MS, Taylor AW, Dawson ED (2011). Identification of *Escherichia coli* O157 by using a novel colorimetric detection method with DNA microarrays. Food Path Dis.

[CR67] Quiñones B, Swimley MS, Narm KE, Patel RN, Cooley MB, Mandrell RE (2012). O-antigen and virulence profiling of Shiga toxin-producing *Escherichia coli* by a rapid and cost-effective DNA microarray colorimetric method. Front Cell Infect Microbiol.

[CR68] Quiñones B, Lee BG, Martinsky TJ, Yambao JC, Haje PK, Schena M (2017). Sensitive genotyping of foodborne-associated human noroviruses and hepatitis A virus using an array-based platform. Sensors.

[CR69] Quiñones B, De Guzman V, Yambao JC, Medin DL, Lee BG (2019) Development of an integrated detection platform for the in-process surveillance of *Listeria* spp. in environmental monitoring samples. Paper presented at the International Association for Food Protection Annual Meeting, Louisville, KY

[CR70] Quiñones B, Lee BG, Yambao JC (2020) *Listeria* detection. United States Patent and Trademark Office, Patent Application Number 16/815,323

[CR71] Rocourt J, Boerlin P, Grimont F, Jacquet C, Piffaretti JC (1992). Assignment of *Listeria grayi* and *Listeria murrayi* to a single species, *Listeria grayi*, with a revised description of *Listeria grayi*. Int J Syst Evol Microbiol.

[CR72] Rodríguez-Lázaro D, Hernández M, Pla M (2004). Simultaneous quantitative detection of *Listeria* spp. and *Listeria monocytogenes* using a duplex real-time PCR-based assay. FEMS Microbiol Lett.

[CR73] Rosimin AA, Kim MJ, Joo IS, Suh SH, Kim KS (2016). Simultaneous detection of pathogenic *Listeria* including atypical *Listeria innocua* in vegetables by a quadruplex PCR method. LWT - Food Sci Technol.

[CR74] Ryu J, Park SH, Yeom YS, Shrivastav A, Lee SH, Kim YR, Kim HY (2013). Simultaneous detection of *Listeria* species isolated from meat processed foods using multiplex PCR. Food Control.

[CR75] Sallen B, Rajoharison A, Desvarenne S, Quinn F, Mabilat C (1996). Comparative analysis of 16S and 23S rRNA sequences of *Listeria* species. Int J Syst Bacteriol.

[CR76] Sayers EW, Cavanaugh M, Clark K, Ostell J, Pruitt KD, Karsch-Mizrachi I (2020). GenBank Nuc Acids Res.

[CR77] Scallan E, Hoekstra RM, Angulo FJ, Tauxe RV, Widdowson MA, Roy SL, Jones JL, Griffin PM (2011). Foodborne illness acquired in the United States-Major pathogens. Emerg Infect Dis.

[CR78] Schrader C, Schielke A, Ellerbroek L, Johne R (2012). PCR inhibitors - occurrence, properties and removal. J Appl Microbiol.

[CR79] Shanks RMQ, Dashiff A, Alster JS, Kadouri DE (2012). Isolation and identification of a bacteriocin with antibacterial and antibiofilm activity from *Citrobacter freundii*. Arch Microbiol.

[CR80] Soriano JM, Rico H, Moltó JC, Mañes J (2001). *Listeria* species in raw and ready-to-eat foods from restaurants. J Food Prot.

[CR81] Sowers BA, Songster MF, Smith KE, Snyder DL, Cook RM (2005) RealTimeDesign™ Software - An Advanced Web-based Program for Real-time PCR Sequence Design. In: 2nd International qPCR Event, Freising-Weihenstephan, Germany

[CR82] Sowers BA, Songster MF, Smith KE, Snyder DL, Cook RM (2006) RealTimeDesign™ - Free Web-based Software For Dual-labeled and Amplifluor® Design In: 20th Anniversary San Diego Conference DNA Probe Technology, San Diego

[CR83] Suh SH, Jaykus LA (2013). Nucleic acid aptamers for capture and detection of *Listeria* spp. J Biotechnol.

[CR84] Suh SH, Dwivedi HP, Choi SJ, Jaykus LA (2014). Selection and characterization of DNA aptamers specific for *Listeria* species. Anal Biochem.

[CR85] Teng J, Yuan F, Ye Y, Zheng L, Yao L, Xue F, Chen W, Li B (2016). Aptamer-based technologies in foodborne pathogen detection. Front Microbiol.

[CR86] The United Fresh Food Safety & Technology Council (2018). Guidance on enviromnental monitoring and control of *Listeria* for the fresh produce industry.

[CR87] Waleh NS, Ingraham JL (1976). Pyrimidine ribonucleoside monophosphokinase and the mode of RNA turnover in *Bacillus subtilis*. Arch Microbiol.

[CR88] Wei S, Daliri EBM, Chelliah R, Park BJ, Lim JS, Baek MA, Nam YS, Seo KH, Jin YG, Oh DH (2019). Development of a multiplex real-time PCR for simultaneous detection of *Bacillus cereus*, *Listeria monocytogenes*, and *Staphylococcus aureus* in food samples. J Food Saf.

[CR89] Williams TR, Marco ML (2014). Phyllosphere microbiota composition and microbial community transplantation on lettuce plants grown indoors. mBio.

[CR90] Williams TR, Moyne AL, Harris LJ, Marco ML (2013). Season, irrigation, leaf age, and *Escherichia coli* inoculation influence the bacterial diversity in the lettuce phyllosphere. PLoS ONE.

[CR91] Woese CR (1987). Bacterial evolution. Microbiol Rev.

[CR92] Yusuf D, Marz M, Stadler PF, Hofacker IL (2010). Bcheck: A wrapper tool for detecting RNase P RNA genes. BMC Genom.

[CR93] Zeinali T, Jamshidi A, Bassami M, Rad M (2017). Isolation and identification of *Listeria* spp. in chicken carcasses marketed in northeast of Iran. Int Food Res J.

[CR94] Zhang Y, Xu CQ, Guo T, Hong L (2018). An automated bacterial concentration and recovery system for pre-enrichment required in rapid *Escherichia coli* detection. Sci Rep.

[CR95] Zoellner C, Ceres K, Ghezzi-Kopel K, Wiedmann M, Ivanek R (2018). Design elements of *Listeria* environmental monitoring programs in food processing facilities: A scoping review of research and guidance materials. Compr Rev Food Sci Food Saf.

